# Analysis of porcine *MUC4 *gene as a candidate gene for prolificacy QTL on SSC13 in an Iberian × Meishan F_2 _population

**DOI:** 10.1186/1471-2156-12-93

**Published:** 2011-10-31

**Authors:** Ingrid Balcells, Anna Castelló, Anna Mercadé, José L Noguera, Amanda Fernández-Rodríguez, Armand Sànchez, Anna Tomàs

**Affiliations:** 1 Departament de Genètica Animal, Centre de Recerca en Agrigenòmica (CRAG), Universitat Autònoma de Barcelona, 08193 Bellaterra, Spain; 2 Servei Veterinari de Genètica Molecular, Universitat Autònoma de Barcelona, 08193 Bellaterra, Spain; 3 Genètica i Millora Animal, IRTA-Lleida, 25198 Lleida, Spain; 4 Departamento de Mejora Genética Animal, SGIT-INIA, 28040 Madrid, Spain; 5 Program Infection and Immunity, FISIB, 07110 Bunyola, Spain

## Abstract

**Background:**

Reproductive traits, such as prolificacy, are of great interest to the pig industry. Better understanding of their genetic architecture should help to increase the efficiency of pig productivity through the implementation of marker assisted selection (MAS) programmes.

**Results:**

The *Mucin 4 *(*MUC4*) gene has been evaluated as a candidate gene for a prolificacy QTL described in an Iberian × Meishan (Ib × Me) F_2 _intercross. For association analyses, two previously described SNPs (DQ124298:g.243A>G and DQ124298:g.344A>G) were genotyped in 347 pigs from the Ib × Me population. QTL for the number of piglets born alive (NBA) and for the total number of piglets born (TNB) were confirmed on SSC13 at positions 44 cM and 51 cM, respectively. The *MUC4 *gene was successfully located within the confidence intervals of both QTL. Only DQ124298:g.344A>G *MUC4 *polymorphism was significantly associated with both NBA and TNB (*P-value *< 0.05) with favourable effects coming from the Meishan origin. *MUC4 *expression level was determined in F_2 _sows displaying extreme phenotypes for the number of embryos (NE) at 30-32 days of gestation. Differences in the uterine expression of *MUC4 *were found between high (NE ≥ 13) and low (NE ≤ 11) prolificacy sows. Overall, *MUC4 *expression in high prolificacy sows was almost two-fold increased compared with low prolificacy sows.

**Conclusions:**

Our data suggest that *MUC4 *could play an important role in the establishment of an optimal uterine environment that would increase embryonic survival during pig gestation.

## Background

Prolificacy traits have been widely explored during the last decades as a potential tool for increasing efficiency of sow productivity in the pig industry. Genetic improvement programmes have achieved moderate gains in prolificacy related traits owing to their low heritability, late expression in life and sex limitation [1]. Increasing knowledge on the genetic architecture of prolificacy traits would provide new tools to improve the efficiency of genetic selection by implementing marker assisted selection (MAS).

So far a relatively low number of quantitative trait loci (QTL) for prolificacy traits reaching the genome-wide significance level have been identified [2,3]. The most significant QTL affecting the number of piglets born alive (NBA) and the total number of piglets born (TNB) were described by Noguera *et al*. [3] in the same resource population as used in the present study, an Iberian (Ib) by Meishan (Me) F_2 _intercross. A number of epistatic QTL were also detected, thus indicating that the genetic architecture of reproductive traits is built as a complex network of interactions throughout the genome. Some of these epistatic QTL were further confirmed and putative candidate interacting genes were identified [4].

Porcine chromosome 13 (SSC13) harbours the most significant QTL for TNB and NBA [3]. The *Mucin 4 *(*MUC4*) gene is located within the confidence interval of prolificacy QTL. Mucins are large heterodimeric glycoproteins commonly located on apical surfaces of many wet-surfaced epithelia that play a key role in the lubrication and protection of the uterine mucosa [5-7]. They have been shown to present anti-adhesive and anti-recognition properties which are necessary to protect the endometrium from the binding and invasion of the trophoectoderm [8,9]. A role of *MUC4 *has been pointed out in rodents and pigs during pregnancy although its expression during the peri-implantational period varies depending on the type of implantation in each species. In mice and rats, which have an invasive type of implantation, *MUC4 *expression is downregulated to generate the receptive state for uterine implantation [8-12]. Conversely, in pigs, where a non-invasive epitheliochorial placental attachment takes place, *MUC4 *is upregulated in the uterus [13]. A protective role has been suggested for *MUC4 *owing to the fact that it is localized on the endometrium epithelium blocking the access of different substrates to the cell surface [14]. The endometrium is then protected from proteolytic activity of porcine conceptus [13] and from microbial invasion [15] resulting in better uterine conditions for embryo development. In pigs, the disruption of the uterine microenvironment could affect embryo viability which could lead to prenatal mortality rates ranging from 20 to 46% [16]. The improvement of the uterine microenvironment would increase embryonic survival and, in consequence, the number of piglets born alive.

In humans, polymorphisms in the *MUC4 *nucleotide sequence have been significantly associated with the development of endometriosis and endometriosis related infertility [17]. However, no association with implantation failure has been detected [18]. In livestock species, the genetic association of *MUC4 *gene variants with reproductive traits has not yet been explored. In pigs, polymorphisms in the *MUC4 *gene were shown to be in linkage disequilibrium with susceptibility/resistance to Enterotoxigenic *Escherichia coli *(ETEC) F4ab/ac infection [19].

In the current study, we have examined the porcine *MUC4 *gene as a functional and positional candidate gene to explain the prolificacy QTL previously identified on SSC13 in the Ib × Me population [3].

## Results

### Refinement of SSC13 QTL for NBA and TNB

The resulting linkage map for SSC13 in the Ib × Me population was as follows (distance is indicated in centimorgan (cM)): S0076 (0.0) - ITIH3 (18.7) - SWR1008 (27.5) - MUC4 (39.1) - SW398 (55.8) - SW2440 (78.5) - SW769 (91.5). The position of the *MUC4 *gene is in agreement with the pig genome sequence (*Sus scrofa*, Ensembl release 64 - September 2011).

**Figure 1:**
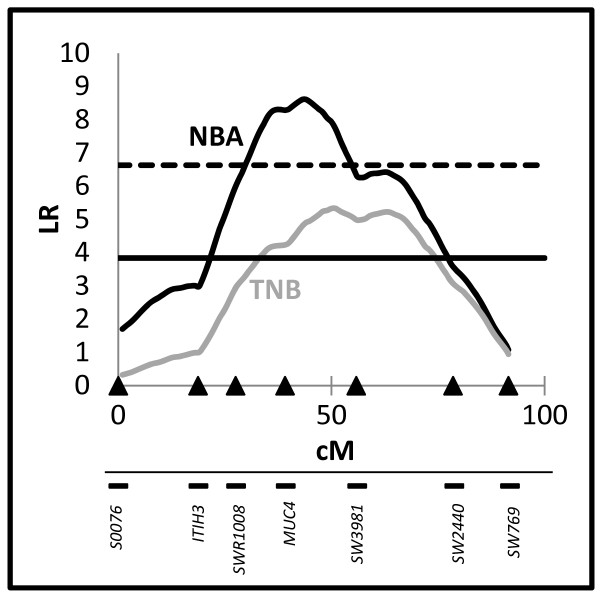
**QTL profiles on SSC13 for NBA and TNB showing the likelihood ratio test statistic**. The horizontal lines set at 3.84 and 6.63 show the 0.05 and the 0.01 significance levels respectively.

A single QTL scan (model 1) was performed on SSC13 for TNB and NBA (Figure 1). As previously described by Noguera *et al*. [3], two significant QTL for NBA and for TNB were identified at positions 44 cM and 51 cM, respectively (Table 1). Significant additive effects were detected for both QTL which determined an increase of 0.65 ± 0.22 piglets per copy for NBA and 0.51 ± 0.22 for TNB. It is noteworthy that the *MUC4 *gene was mapped within the confidence interval (CI) of both QTL.

**Table 1:** Results of QTL analyses, association tests and marker assisted association tests for prolificacy traits

	QTL model (model 1)			*MUC4 *association (model 2)		QTL + *MUC4 *association model (model 3)^1^			
			
Trait	Pos. (c.i)^2^	*P*-QTL	a_QTL _(SE)^3^	*P*-MUC4	a _MUC4 _(SE)^4^	Pos.^5^	*P*-QTL + MUC4 (DQ124298:g.344A>G)	*P*- QTL	*P*-MUC4 (DQ124298:g.344A>G)
NBA	44 (22-77)	0.003	0.65 (0.22)	0.006	0.74 (0.27)	63	0.003	0.062	0.007
TNB	51 (33-74)	0.021	0.51 (0.22)	0.037	0.57 (0.27)	64	0.019	0.061	0.039

^1^*P-values *obtained with the model that includes the QTL and the DQ124298:g.344A>G *MUC4 *SNP effects; each effect(s) was tested by removing it from the null model; ^2^QTL position (in cM); c.i = confidence interval; ^3^QTL additive effect; SE = standard error; ^4^DQ124298:g.344A>G *MUC4 *SNP additive effect; ^5^QTL position in cM when corrected by the DQ124298:g.344A>G *MUC4 *SNP effect.

### Candidate gene association analyses

Allele frequencies for DQ124298:g.243A>G and DQ124298:g.344A>G SNPs in the Ib × Me population are shown in Table 2. Associations between *MUC4 *polymorphisms and reproductive traits were tested with a standard animal model (model 2). No significant associations were found for the DQ124298:g.243A>G SNP and the reproductive traits recorded (data not shown). For the DQ124298:g.344A>G SNP, the results supported significant additive effects between this SNP and NBA and TNB traits (*P-value *< 0.05, Table 1). The additive substitution effect for DQ124298:g.344G SNP was estimated to be 0.74 ± 0.27 for NBA (*P-value *= 0.006) and 0.57 ± 0.27 for TNB (*P-value *= 0. 0.037). In both cases, the G allele coming from the Me breed had a favourable effect. Note that DQ124298:g.344A>G SNP had a larger effect on NBA than on TNB (*P-value *= 0.006 for NBA and *P-value *= 0.037 for TNB).

**Table 2:** Allelic frequencies of the DQ124298:g.243A>G and DQ124298:g.344A>G *MUC4 *polymorphisms in the Me × Ib F_2 _population

		DQ124298:g.243			DQ124298:g.344		
		
		n	A	G	n	A	G
F_0 _	♀ Meishan	18	0	1	18	0	1
	♂ Iberian	3	0.50	0.50	3	0.50	0.50

F_1_		120	0.32	0.68	123	0.18	0.82

F_2_		206	0.20	0.80	202	0.29	0.71

The results obtained with association studies must be interpreted with caution due to the fact that the extensive linkage disequilibrium described in F_2 _crosses can bias the estimations. For this reason, in order to detect possible false positives obtained in association studies with DQ124298:g.344A>G SNP, a marker assisted association test (MAAT) including the QTL effect in the association test [20] was performed with model 3. Results from the MAAT are summarized in Table 1. The DQ124298:g.344A>G SNP effect on NBA and TNB remained significant when the QTL effect was considered which is in agreement with the association studies analyses. Nevertheless, the significance of the QTL disappeared when the DQ124298:g.344A>G SNP was included in the model. MAAT shows that the DQ124298:g.344A>G SNP genotype explains better the effects on NBA and TNB and confirms the association detected with model 2.

### Expression analysis of the porcine *MUC4 *gene

**Figure 2:**
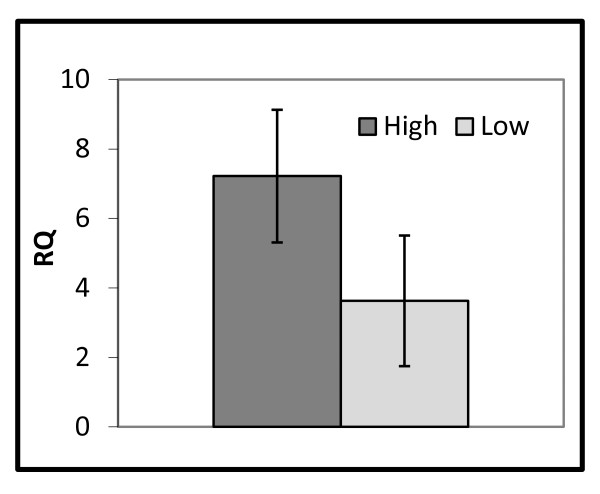
**Relative quantification (RQ) of the porcine *MUC4 *gene in the uterus**. *MUC4 *expression in the uterus was measured in 36 F_2 _sows that were classified into two groups according to the number of embryos (NE) at the sacrifice day (30-32 days of gestation): low (NE ≤ 11) and high (NE ≥ 13).

In order to determine whether *MUC4 *expression could affect prolificacy related traits, we analysed the uterine expression profile of porcine *MUC4 *in sows that differed in the number of embryos (NE) at 30-32 days of gestation using qPCR. At this time of gestation, the embryo is already attached to the endometrium and the foetal survival rate will be an indication of the final litter size [1]. For this reason, NE was measured as an estimation of prolificacy. Results showed that mRNA expression levels of *MUC4 *gene were suggestively greater in high (NE ≥ 13, n = 16, mean relative expression = 7.22) than in low (NE ≤ 11, n = 20, mean relative expression = 3.63) prolificacy sows (*P-value *= 0.07, Figure 2), reaching almost a two-fold increase in the high prolificacy group.

## Discussion

Statistical association between the DQ124298:g.344A>G mutation in the porcine *MUC4 *gene and prolificacy related traits has been reported. No association was found, however, between the DQ124298:g.243A>G SNP and the prolificacy related traits although this SNP is located 100bp upstream of the DQ124298:g.344A>G mutation. The difference in association analysis detected between both *MUC4 *SNPs indicates that they are not in linkage disequilibrium. The *MUC4 *gene successfully mapped within the confidence interval of the SSC13 QTL affecting NBA and TNB traits previously described by Noguera *et al*. [3] using the same resource population. The favourable effect of DQ124298:g.344A>G SNP was inherited from the Me maternal breed, as expected, since Me is more prolific than Ib. The effect of the DQ124298:g.344A>G *MUC4 *SNP was stronger for NBA than for TNB suggesting that the *MUC4 *gene could be related to the embryonic survival in the uterus. This hypothesis is supported by the differential expression of the *MUC4 *gene found in the uterus at 30-32 days of gestation, where the expression was two-fold higher in high than in low prolificacy sows. Nevertheless, further studies must be performed in other unrelated porcine populations to validate the association which was obtained in this study.

*MUC4 *has been identified as a potential regulator of placentation in pigs [21]. In porcine uterine surface epithelium, *MUC4 *gene expression increases during the first 18 days of gestation [13] whereas in rats a reduction in *MUC4 *gene expression in the uterus is associated with the period of implantation [12]. Differences in *MUC4 *expression profiles could be explained by the different placentation types of rodents and pigs. Pigs have a non-invasive type of placentation and MUC4 is thought to play a role in protecting the uterus from erosion by the embryo [13,15]. MUC4 and MUC1 are the major mucin proteins expressed in the endometrial epithelium [22,23]. Human *MUC1 *and *MUC4 *present highly polymorphic sites with a variable number of tandem repeats (VNTRs). *MUC1 *VNTR variants have been related to alterations in both T-antigen presentation and in the local immune response in cancer [17,24,25]. These results suggest that mucins may play a role in the immunological processes that take place during the implantation period essential to ensure the correct establishment of maternal-foetal tolerance [26].

## Conclusions

*MUC4 *polymorphism DQ124298:g.344A>G is associated with litter size in the Ib × Me population and, moreover, *MUC4 *is differentially expressed regarding the number of embryos in uterus at 30 days of gestation. These results suggest that *MUC4 *may participate in the establishment of an optimal uterine environment essential for adequate embryo development during the early stages of gestation and increase litter size in pigs.

## Methods

### Animal material and phenotypic measurements

An F_2 _population was generated by crossing 3 Ib males from the Guadyerbas line (Dehesón del Encinar, Toledo, Spain) with 18 Me females (Domaine du Magneraud, INRA, France). A total of 8 boars and 97 sows from the F_1 _generation were mated to obtain the F_2 _progeny in the Nova Genètica S.A experimental farm (Lleida, Spain). All animals were obtained according to the European animal experimentation ethics law approved by the Ethical and Care Committee at IRTA.

Measurements of sow reproduction traits including TNB and NBA were recorded in 255 F_2 _sows during 4 successive parities. In the fifth parity, sows were slaughtered at 30-32 days of gestation, when the uterus samples were recollected and the number of embryos (NE) was recorded.

### Genotyping of *MUC4 *polymorphisms

Two *MUC4 *polymorphisms described by [19], two A-to-G substitution polymorphisms at positions 243 and 344 of intron 17 (GenBank accesion number DQ124298), were genotyped in our Ib × Me population by pyrosequencing [27] in a PSQ HS 96 system (Pyrosequencing AB, Uppsala, Sweden). Two PCR amplifications, one for each SNP, were carried out in a 25 μl total volume that included 1.5 mM of MgCl_2_, 200 μM of each dNTP, 0.75 U of TaqGold DNA polymerase (Applied Biosystems), 320 nM of each primer (Table 3) and 40 ng of DNA. The thermal profile was 95°C for 10 min, 40 cycles at 94°C for 30 s, 58°C for 45 s and 72°C for 1 min and a final extension step of 15 min at 72°C. A multiplex pyrosequencing reaction was performed with 5 μl of each PCR with the primers described in Table 3.

**Table 3:** List of primer sequences used for typing *MUC4 *polymorphisms and quantitative PCR

Application	SNP/Gene	Primer Sequence (5' →3')	Length (bp)
**Pyrosequencing PCR**	DQ124298:g.243A>G	F: *tggtgctacccccagatttg	
		R: gttgtgtccaccccttacccttat	195
		P: gtcccctctcccaggta	
	
	DQ124298:g.344A>G	F: *gtggccctcagtcactagagt	
		R: cgaagttgtgaaaggaagacag	258
		P: ttggggttggggcag	

**qPCR**	*MUC4*	F: atgggcttctccagtggagat	66
		R: tctcccacactggctgcaa	

* 5' Biotin labelled, F forward primer, R reverse primer, P pyrosequencing primer

### QTL and association analyses

The linkage map of porcine chromosome 13 (SSC13) was constructed with the *Build *option of CRIMAP 2.4 software [28]. Overall, seven markers were used: five microsatellites that had been previously described by [3], one SNP at the *ITIH3 *gene (dbSNP accession number ss315834911) and the DQ124298:g.344A>G *MUC4 *SNP described by [19].

Three models were used to analyse the Ib × Me F_2 _population data: (1) a QTL model, (2) an association model and (3) a QTL + association model to perform the marker assisted association test (MAAT) proposed by [20].

First, one dimensional QTL mapping was performed with model 1.

$$y_{ijk}=H_{i}+O_{j}+u_{k}+p_{k}+C_{a}a+e_{ijkl}\;\left(\text{model 1}\right)$$

where *y_ijk _*was the phenotypic data vector for NBA or TNB; *H_i _*and *O_j _*were the fixed effects for year-season and parturition order, respectively; *u_k _*was the random polygenic effect of each individual; *p_k _*was the environmental permanent effect of the sow; *a *was the QTL additive effect; *C_a _*was the probability of the individual being homozygous for Ib alleles minus the probability of being homozygous for Me alleles at the QTL position of interest; and *e_ijkl _*was the random residual term. The dominance effect was not included in the model because the likelihood ratio test performed indicated that a model with only additive QTL effect fitted better.

Second, association analyses were performed with the *MUC4 *SNPs (DQ124298:g.243A>G and DQ124298:g.344A>G) with a standard animal model (model 2).

$$y_{ijk}=H_{i}+O_{j}+u_{k}+p_{k}+\underset{k}{\sum }\lambda_{ik}a_{k}+e_{ijkl}\;\left(\text{model 2}\right)$$

where *y_ijk _*was the vector containing the phenotypic data for NBA and TNB and *λ_ik _*was a variable that indicated the number of copies (0, 1, or 2) of the *k*^th ^allele presented by each individual.

Finally, a combined QTL + association model (model 3) was used to consider the extensive linkage disequilibrium present in F_2 _population. Only the DQ124298:g.344A>G SNP was considered because significant results were obtained in model 2.

$$y_{ijk}=H_{i}+O_{j}+u_{k}+p_{k}+C_{a}a+\underset{k}{\sum }\lambda_{ik}a_{k}+e_{ijkl}\;\left(\text{model 3}\right)$$

All analyses were performed with Qxpack software [29]. QTL scans were analysed every cM and nominal *P-values *were calculated with the maximum likelihood ratio test, assuming a χ^2 ^distribution of the likelihood ratio test with degrees of freedom calculated as the difference between the number of parameters in the alternative and in the null models.

### Expression analysis of the *MUC4 *gene

Uterine expression of the *MUC4 *gene was measured by reverse transcription quantitative real time PCR (RT-qPCR). Total RNA from uterus samples was extracted by means of the RiboPure™ kit (Ambion, Applied Biosystems). One microgram of total RNA in 40 μl reaction was reverse transcribed with the High Capacity cDNA Transcription Kit (Applied Biosystems). Primers for *MUC4 *(Table 3) were designed with Primer Express^® ^2.0 software (Applied Biosystems, Warrington, UK). *Sus scrofa hypoxanthine phosphoribosyltransferase 1 *(*HPRT1*) was used as a reference gene for normalization [for primer sequences see 30]. qPCR reactions were performed in triplicate in a 20 μl final volume including 2X FastStart SYBR Green Master (Roche), 0.3 μM of each primer and 5 μl of the cDNA diluted twenty times on an ABI PRISM^® ^7900HT sequence detection system (Applied Biosystems, Warrington, UK). Thermal conditions were 95°C for 10 min followed by 40 cycles of 95°C for 15 s and 60°C for 1 min. Dissociation curve analyses were performed in order to detect unspecific amplifications. PCR efficiencies of *MUC4 *and *HPRT1 *genes were calculated to validate the use of the 2^-ΔΔCt ^method [31]. The *MUC4 *gene expression level was measured individually in 36 Ib × Me F_2 _sows from 32 different litters that were classified into two groups according to the NE at slaughter (30-32 days of pregnancy): low (NE ≤ 11, n = 20) and high (NE ≥ 13, n = 16). Gene expression data were log_10 _transformed and analysed by a t-test using the Statistical Analysis System (Statistics, V 9.1.3; SAS Institute, Inc., Cary, NC). The significance threshold was set at α < 0.05.

## Authors' contributions

IB carried out qPCR experiments, analysed and interpreted the data and prepared the manuscript. AC performed the genotyping task and revised the manuscript. AM designed the genotyping protocol. JLN participated in the design of the study and coordinated it. AF participated in sample collection and helped with the critical revision of the manuscript. AS conceived the study, participated in the design and the supervision of the study and revised the draft. AT supervised the study, helped to draft the manuscript and revised it. All authors read and approved the final manuscript.
